# Effect of parental physiological conditions and assisted reproductive technologies on the pregnancy and birth outcomes in infertile patients

**DOI:** 10.18632/oncotarget.12553

**Published:** 2016-10-10

**Authors:** Xinqi Zhong, Jianqiao Liu, Qiliang Cui, Shaozhen Liang, Yuanqing Lin, Haiying Liu, Qiyi Zeng

**Affiliations:** ^1^ Department of Pediatrics, Zhujiang Hospital, Southern Medical University, Guangzhou, Guangdong, China; ^2^ Department of Pediatrics, the Third Affiliated Hospital of Guangzhou Medical University, Guangzhou, Guangdong, China; ^3^ Department of Reproductive Medicine, the Third Affiliated Hospital of Guangzhou Medical University, Guangzhou, Guangdong, China

**Keywords:** birth defects, assisted reproductive technologies, pregnancy, infertile, birth outcome

## Abstract

Assisted reproductive technologies (ART) are widely used to treat infertility. Emerging evidence suggested that ART was associated with perinatal or neonatal problems, however, little is known about the ART related risk factors. Here using 21136 ART cases, we determined the impacts of parental physiological conditions in the ART mediated pregnancy outcomes. In addition, we further evaluated the effects of three different ART methods (frozen-thawed embryo transfer [FET], in vitro fertilization [IVF] and intracytoplasmic sperm injection [ICSI]) in the pregnancy and birth outcomes in ART mediated pregnancy. Our data revealed that older parental age increases the risks of abortion, preterm birth and low body weight birth. Higher maternal BMI (Body mass index) level correlates with higher abortion rate. Moreover, pregnancy with multiple fetuses has severer adverse outcomes compared to singleton pregnancy. Among the three ART methods, ICSI is associated with lower ratios of ectopic pregnancy, abortion and deformity compared to FET and IVF. Our study revealed new clinical insights into the ART related risk factors and suggested that both the parental physiological conditions and ART methods should be evaluated to develop better ART mediated infertility treatments.

## INTRODUCTION

ART mediated infertility treatments have brought tremendous benefits to the infertile patients [1]. However, emerging perinatal or neonatal problems have been reported to be associated with ART [2–7]. Recent studies revealed ART as an independent factor that contributes to the poor maternal and neonatal outcomes [4–6, 8–10]. For example, a systematic review of 25 studies suggested that, infants born with ART have a significant 30-40% increased risk of birth defects compared to those born in spontaneous pregnancy [8]. In addition, ART is associated with increased risk of as low birth weight (LBW) [2, 3], preterm birth (PTB) [3], small gestational age [11], and childhood cancers [12].

Currently little is known about the factors associated with ART risks. Some studies showed that the social statuses, pregnancy related characteristics and medical conditions are different between the ART treated mothers and the non-ART treated mothers [13–15], however, whether the above difference contributes to the increased risk of adverse pregnancy outcomes is still inconclusive. In addition, different assisted reproduction techniques might have different successful rates and risks, while at this point whether different ART techniques are associated with different risks of bad clinical outcomes remains to be characterized.

Characterization of the physiological and technical factors that associated the increased risks of ART is crucial for the development of better ART treatment strategies for infertile patients. In this study, we studied the associations between parental physiological conditions and pregnancy/birth outcomes in 21136 ART cases. In addition, we further compared the pregnancy/birth outcomes in ART cases that performed with three different ART methods (FET, IVF, and ICSI). Our data provided novel insights into the ART related risk factors and could potentially be used for clinical guidelines in ART treatment for infertile patients.

## MATERIALS AND METHODS

### Study population

ART cases performed between Jan 2010 and Dec 2014 in Reproductive Medicine Center of Guangzhou Medical Centre for Critical Pregnant Women in Guangzhou Institute of Obstetrics and Gynecology, Guangzhou, China, were used for this study. 21136 ART cases that had detail information about patient physiological conditions, treatment processes, birth outcomes were included for the study. Patients with other major health problems, without reliable information, did not complete follow up and patients who did not sign the consents form were excluded. The ART cases that used donor ovocytes or sperms were excluded. All the patients are Asian. The study protocol was approved by the Institutional Ethic Committee of the Hospital. Written informed consents were obtained from all patients before collecting the clinical information.

### Data collection

Patient and pregnancy data were recorded by the hospital specialists from patient's first consultation to final neonatal birth. Data were retrieved from the hospital records to study the ART related risk factors. Patients’ physiological conditions, including parental ages, infertile types and years, mother's BMI, pregnancy outcomes (ectopic pregnancy, abortion, premature birth, deformity and low birth weight infants) were used to evaluate the role of physiological conditions in ART mediated pregnancy outcomes. In addition, the clinical outcomes of ART cases performed using three different ART methods including IVF, ICSI and FET were investigated to determine the effect of different ART methods in pregnancy and birth outcomes.

### Definitions of birth outcomes

Ectopic pregnancy refers to the pregnancy that occurs outside the uterus. Abortion means the pregnancy is terminated during the first 28 weeks of pregnancy. Preterm birth is defined as a premature birth that takes place three weeks before the due day (before the 37^th^ week of pregnancy). Deformity means the fetus that has deformed body parts. Low body weight birth is defined as a birth in which the infant's weight is less than 2,500g.

### Statistical analysis

SPSS17.0 software was used for the statistics analysis. Measurement data were presented as mean ± standard deviation, and enumeration data were presented by frequency. Pearson χ2 test or χ2 test with continuity correction was used for the comparison of enumeration data. Chi-square test of four-fold table and contingency table were used for the analysis of two groups and multiple groups of enumeration data, separately. A *p* value < 0.05 was considered as statistically significant.

## RESULTS

### The pregnancy outcome in the ART cases

In the 21136 ART cases, the average maternal age was 32.0±4.4 (range: 20-49), average paternal age was 34.5±5.3 (range: 21-78), and the average infertile years was 4.9±3.3 (range: 1-23). After ART treatments, 10139 patients successfully achieved pregnancy, accounting for 48.0% of the total cases. Of the 10139 pregnancies, there were 9819 cases (96.8% of total pregnancies) of intrauterine pregnancies, 283 cases (2.8%) of ectopic pregnancies and 37 cases (0.4%) of simultaneously intrauterine and ectopic pregnancies. There were 8066 cases of successful delivery (38.2% of total ART cases), including 5470 (67.8%) singletons and 2596 (32.2%) multiple births. Totally 10695 babies (5748 male and 4947 female) were born from the 8066 deliveries, among those there were 3214 cases of low body weight and 116 cases of deformities.

### The clinical factors affecting the successful rate of pregnancy

To determine the ART risk factors, we first investigated the association between the patients’ physiological conditions and the pregnancy outcomea in the 21136 ART cases. As shown in Table 1, the patients with primary infertility had significant higher ratio (49.1%) of successful pregnancy than those with secondary infertility (46.8%). The pregnancy rate was significant higher in families with only one infertile parent compared to those families in which both parents were infertile. In addition, older parental age, higher maternal BMI and longer infertile time were significantly associated with lower rates of pregnancy.

**Table 1:** Influence of parental physiological conditions in ART clinical pregnancy

	Pregnant		Not pregnant		Total	χ2	p
	n	%	n	%			
Infertile Types						10.86	0.001
Primary	5240	49.1	5434	50.9	10674		
Secondary	4899	46.8	5563	53.2	10462		
Infertile Gender						20.57	<0.001
Male	1459	49.1	1513	50.9	2972		
Female	6940	48.6	7332	51.4	14272		
Both	1740	44.7	2152	55.3	3892		
Maternal Age						452.16	<0.001
<35	8002	52.5	7235	47.5	15237		
≥35	2137	36.2	3762	63.8	5899		
Maternal BMI						20.5	0.000
<24	7920	49.8	7972	50.2	15892		
≥24	1738	45.7	2061	54.3	3799		
Paternal Age						216.82	<0.001
<35	6073	52.6	5477	47.4	11550		
≥35	4066	42.4	5520	57.6	9586		
Infertile Years						146.20	<0.001
<2	1229	52.7	1103	47.3	2332		
2-3	3247	51.6	3047	48.4	6294		
4-5	2547	49.2	2626	50.8	5173		
≥6	3116	42.5	4221	57.5	7337		
Total	10139		10997		21136		

### Association between maternal age and birth outcomes

Next we investigated the potential association between maternal age and the pregnancy/birth outcomes (Table 2) in the ART cases. The ratios of different pregnancy and birth outcomes (ectopic pregnancy, abortion, preterm birth, low body weight birth and deformity) were evaluated in two different maternal age groups. As shown in Table 2, the ratio of abortion in older age (≥35) group was 21.9%, significantly higher than the ratio (13.8%) in the younger age ( < 35) group. While the ratios of low body weight and preterm birth were significantly lower in the older age group. There was no statistically significant difference in the ratio of ectopic pregnancy and deformity in the two different age groups.

**Table 2:** Influence of maternal age on pregnancy and birth outcome

		<35		≥35		total	χ2	p
		n	%	n	%			
ectopic pregnancy	yes	248	3.1	72	3.4	320	0.40	0.526
	no	7754	96.9	2065	96.6	9819		
	total	8002	100.0	2137	100.0	10139		
abortion	yes	1106	13.8	468	21.9	1574	83.93	0.000
	no	6896	86.2	1669	78.1	8565		
	total	8002	100.0	2137	100.0	10139		
preterm birth	yes	1716	26.3	371	23.8	2087	4.40	0.036
	no	4799	73.7	1191	76.2	5990		
	total	6515	100.0	1562	100.0	8077		
deformity	yes	89	1.4	18	1.2	107	0.42	0.515
	no	6434	98.6	1541	98.8	7975		
	total	6523	100.0	1559	100.0	8082		
low body weight birth	yes	1830	28.1	363	23.3	2193	14.91	0.000
	no	4680	71.9	1197	76.7	5877		
	total	6510	100.0	1560	100.0	8070		

### The influence of maternal BMI levels on the birth outcomes

The parents were separated into two different groups (BMI high and BMI low) to study the association between maternal BMI levels and pregnancy/birth outcomes (Table 3). The ratio of abortion in BMI high (≥24) group was 18.2%, which was significantly higher compared to 14.9% in BMI low ( < 24) group. There's no statistically significant difference in ectopic pregnancy, preterm birth, deformity and low body weight between the BMI high and low groups.

**Table 3:** Influence of maternal BMI on pregnancy and birth outcome

		<24		≥24		total	χ2	p
		n	%	n	%			
ectopic pregnancy	yes	249	3.1	47	2.7	296	0.93	.336
	no	7671	96.9	1691	97.3	9362		
	total	7920	100.0	1738	100.0	9658		
abortion	yes	1180	14.9	317	18.2	1497	12.14	.000
	no	6740	85.1	1421	81.8	8161		
	total	7920	100.0	1738	100.0	9658		
preterm birth	yes	1645	25.9	357	26.6	2002	0.28	.596
	no	4715	74.1	987	73.4	5702		
	total	6360	100.0	1344	100.0	7704		
deformity	yes	86	1.4	18	1.3	104	0.00	.981
	no	6282	98.6	1323	98.7	7605		
	total	6368	100.0	1341	100.0	7709		
low body weight birth	yes	1759	27.7	349	26.0	2108	1.47	.225
	no	4598	72.3	991	74.0	5589		
	total	6357	100.0	1340	100.0	7697		

### The influence of fetus numbers on the birth outcomes

ART treatments often result in multiple fetuses in the pregnancy, we further determined the association between fetus numbers and the pregnancy/birth outcomes in our ART cases (Table 4). The ratio of ectopic pregnancy in patients with multiple fetuses was 1.3%, significantly lower than 4.6% in patients with singleton. The ratio of abortion was also significantly lower in patients with multiple fetus (10.2%) compared to those with singleton (21.1%). On the other hand, in the patients with multiple fetuses, the ratios of preterm birth, deformity and low body weight birth were 49%, 2.0% and 57.5%, which were significantly higher than the ratios in the patients with singleton (9.2%, 0.9% and 6.7%, respectively).

**Table 4:** Influence of fetus number on pregnancy and birth outcome

		singleton		multiple fetuses		total	χ2	p
		n	%	n	%			
ectopic pregnancy	yes	216	4.6	39	1.3	255	62.40	0.000
	no	4440	95.4	2940	98.7	7380		
	total	4656	100.0	2979	100.0	7635		
abortion	yes	982	21.1	304	10.2	1286	153.71	0.000
	no	3674	78.9	2675	89.8	6349		
	total	4656	100.0	2979	100.0	7635		
preterm birth	yes	310	9.2	1251	49.0	1561	1184.00	0.000
	no	3058	90.8	1303	51.0	4361		
	total	3368	100.0	2554	100.0	5922		
deformity	yes	32	0.9	51	2.0	83	11.58	0.001
	no	3340	99.1	2501	98.0	5841		
	total	3372	100	2552	100	5924		
low body weight birth	yes	226	6.7	1467	57.5	1693	1828.62	0.000
	no	3136	93.3	1086	42.5	4222		
	total	3362	100.0	2553	100.0	5915		

### The influence of different ART methods on the birth outcomes

We next investigated whether different ART treatment methods could affect the pregnancy outcomes. There different ART methods were used for the treatment of infertile patients. As shown in Table 5, the ratios of ectopic pregnancy, abortion and deformity were significant lower in the ICSI group compared to the FET and IVF groups. On the other hand, the ratio of low body weight birth was significantly lower in the FET group compared to the other two groups. There's no statistically significant difference in the ratio of preterm birth in the three groups.

**Table 5:** Influence of different ART treatments on the pregnancy and birth outcome

		FET		IVF		ICSI		total	χ2	p
		n	%	n	%	n	%			
ectopic pregnancy	yes	94	3.2	204	3.4	22	1.8	320	9.49	0.009
	no	2871	96.8	5723	96.6	1225	98.2	9819		
	total	2965	100.0	5927	100.0	1247	100.0	10139		
abortion	yes	493	16.6	916	15.5	165	13.2	1574	7.77	0.021
	no	2472	83.4	5011	84.5	1082	86.8	8565		
	total	2965	100.0	5927	100.0	1247	100.0	10139		
preterm birth	yes	579	25.1	1251	26.4	257	25.0	2087	1.81	0.406
	no	1728	74.9	3490	73.6	772	75.0	5990		
	total	2307	100.0	4741	100.0	1029	100.0	8077		
deformity	yes	42	1.8	57	1.2	8	0.8	107	7.21	0.027
	no	2268	98.2	4687	98.8	1020	99.2	7975		
	total	2310	100.0	4744	100.0	1028	100.0	8082		
low body weight birth	yes	533	23.1	1386	29.3	274	26.6	2193	29.80	0.000
	no	1772	76.9	3349	70.7	756	73.4	5877		
	total	2305	100.0	4735	100.0	1030	100.0	8070		

## DISCUSSION

More and more evidence suggested that ART is associated with bad pregnancy and birth outcomes, however, little is known about the factors that are correlated with the pregnancy and birth outcomes in the ART treated patients. In this study, we studied the potential ART risk related factors with a total of 21136 ART cases in our hospital from 2010 to 2014. ART is associated with higher ratios of preterm birth (25.8%) and low body weight birth (27.2%) (Table 2) than the ratios (4.37% and 6.1%) reported in natural pregnancies [16, 17]. The ratio of deformity in our ART cases was 1.32% (Table 2), which is very similar to the deformity ratio (1.35%) in the natural pregnancies in China [18].

We then studied the factors that are associated with successful pregnancy and revealed that primary infertility patients have better chance of pregnancy than the secondary infertility patients. In addition, patients with older age, higher BMI and longer infertile time have less ratio of pregnancy after ART treatments. This suggested that the successful rate of pregnancy in ART treatment patients is affected by different parental physiological conditions. Previously, it was reported that the successful rate of ART in ≥40 years old patients was significantly lower than patients younger than 40 [19–21]. In our study, we used 35 years old as the maternal age cutoff because 25-35 years is the optimal age for female to have babies. Our result also revealed that advanced maternal age has negative impact on ART mediated pregnancy. In addition, our results were consistent with the previous reports that obesity increases the risk of abortion in ART pregnancy [22, 23]. Old maternal age and high BMI might be associated with increased chance of ovarian dysfunction; longer duration of infertility might cause stronger pelvic adhesions, therefore decreasing the chance of successful pregnancy.

We next studied the influence of maternal physiological conditions in the birth outcomes in the ART mediated pregnancies. We found that older maternal age was associated with higher ratio of abortion, while interestingly, the ratios of some other bad birth outcomes including low body weight and preterm birth were significantly lower in the older patients. Similarly, higher maternal BMI level and longer duration of infertility were significantly associated with higher chance of abortion but lower ratio of low body weight birth in the ART cases. Older maternal age, higher maternal BMI, longer duration of infertility are usually associated with weakened ovarian function, mis-regulated endometrial morphology and function, increased risks of chromosomal aberrations and decreased uterine receptivity, creating an unfavorable environment for the baby growth [24, 25]. Therefore, the chance for the babies with minor defects (preterm birth and low body weight) to survive during pregnancy decreases, resulting in higher ratio of abortion and accordingly lower ratios of preterm birth and low body weight birth. All these data suggested that the optimal maternal physiological conditions are critical for successful pregnancies and good birth outcomes in the ART treated patients.

In addition to the parental physiological conditions, different ART methods can also affect the pregnancy and birth outcomes in the infertile patients. In this study, we evaluated the effect of three different ART methods, including FET, IVF and ICSI, in the birth outcomes in our ART cases. It has been controversial about the whether there is significant difference in the clinical outcomes between the frozen embryo transfer and fresh embryo transfer ART methods. For example, a recent study suggested that the fresh embryo transfer methods had better pregnancy outcomes than the frozen embryo transfer method [26], while others revealed that FET could reduce the ovarian hyperstimulation syndrome risks and improve birth outcomes [27–29]. In our study, we performed a comprehensive analysis of multiple aspects of birth outcomes and found that FET treatment is associated with significantly lower ratio of low body weight birth but higher ratio of deformity compared to the fresh embryo transfer treatments. Our results suggested that the frozen-thaw process could have both positive and negative effects on the birth outcomes, which could partially explain the controversies about the role of embryo frozen in ART because different early studies only investigated certain aspects of the birth outcomes [26–29].

We also compared the birth outcomes in the two fresh embryo transfer ART methods (IVF and ICSI). Our data revealed that patients treated with ICSI have significantly lower ratios of ectopic pregnancy, abortion and deformity, suggesting that ICSI is associated with better birth outcomes than IVF and FET. ICSI is a ART method in which a single function sperm is injected into the cytoplasm of the egg for the fertilization, and is commonly used to treat the infertile male that have too few motile and morphologically normal sperm [30, 31]. Our finding revealed that ICSI is associated with better birth outcomes compared to conventional IVF, which is consistent with conclusion from a recent Norwegian national population-based cohort study [32].

There are limitations in this study: first, all the study subjects were from the same hospital, the successful rate of pregnancy might be affected by the doctors’ medical skills and the hospital's medical facility conditions. Second, when comparing the pregnancy outcomes in ART mediated pregnancies to the natural pregnancies, we used the published natural pregnancy data, the natural pregnancy data from the same hospital will be better for the comparison.

Overall, we revealed that the parental age, maternal BMI, fetus numbers and different ART methods have significant impacts on the clinical pregnancy and birth outcomes in the ART treated infertile patients. Our study provided novel insights into the ART related risk factors and could serve as clinical guidelines to prevent and reduce the risk of bad outcomes in the ART mediated infertile treatments.
